# The Role of Histology-Agnostic Drugs in the Treatment of Metastatic Castration-Resistant Prostate Cancer

**DOI:** 10.3390/ijms23158535

**Published:** 2022-08-01

**Authors:** Giovanni Maria Iannantuono, Francesco Torino, Roberto Rosenfeld, Simona Guerriero, Manuela Carlucci, Stefano Sganga, Barbara Capotondi, Silvia Riondino, Mario Roselli

**Affiliations:** Medical Oncology Unit, Department of Systems Medicine, University of Rome Tor Vergata, Via Montpellier 1, 00133 Rome, Italy; gmiannantuono@gmail.com (G.M.I.); torino@med.uniroma2.it (F.T.); roberto.rosenfeld88@gmail.com (R.R.); simonaguerriero46@gmail.com (S.G.); manucarlucci5@gmail.com (M.C.); stefano.sganga@gmail.com (S.S.); barbara.capotondi@gmail.com (B.C.); silvia.riondino@uniroma2.it (S.R.)

**Keywords:** prostate cancer, histology-agnostic, dMMR, MSI-H, TMB-H, NTRK, BRAF V600E

## Abstract

Precision medicine has opened up a new era in the development of anti-cancer agents that is focused on identifying biomarkers predictive of treatment response regardless of tumor histology. Since 2017, the Food and Drug Administration has approved six drugs with histology-agnostic indications: pembrolizumab (both for tumors with the mismatch-repair deficiency (dMMR)/high microsatellite instability (MSI-H) phenotype and for those with the high tumor mutational burden (TMB-H) phenotype), dostarlimab (for dMMR tumors), larotrectinib and entrectinib (for tumors harboring neurotrophic tyrosine receptor kinase (NTRK) fusions), and the combination of dabrafenib plus trametinib (for BRAF V600E-mutated tumors). The genomic alterations targeted by these antineoplastic agents are rare in metastatic castration-resistant prostate cancer (mCRPC). Furthermore, only a small number of mCRPC patients were enrolled in the clinical trials that led to the approval of the above-mentioned drugs. Therefore, we critically reviewed the literature on the efficacy of histology-agnostic drugs in mCRPC patients. Although the available evidence derives from retrospective studies and case reports, our results confirmed the efficacy of pembrolizumab in dMMR/MSI-H mCRPC. In contrast, few data are available for dostarlimab, larotrectinib, entrectinib, and dabrafenib-trametinib in this subset of patients. Large, multi-institutional registries aimed at collecting real-world data are needed to better comprehend the role of tissue-agnostic drugs in mCRPC patients.

## 1. Introduction

Prostate cancer (PCa) represents the second most frequently diagnosed tumor and the fifth leading cause of cancer death among men worldwide [1]. Although the treatment paradigm of metastatic PCa has profoundly evolved in recent years, androgen deprivation therapy (ADT) with medical or surgical castration remains the cornerstone of PCa management [2]. Nonetheless, the majority of men affected by advanced PCa develop a resistance to ADT within the first two years of therapy and progress to metastatic castration-resistant prostate cancer (mCRPC) [3]. Several anti-cancer agents are now available for mCRPC, including cytotoxic chemotherapy (docetaxel [4] and cabazitaxel [5]), androgen receptor signaling inhibitors (ARSIs) (abiraterone acetate [6] and enzalutamide [7]), poly ADP-ribose polymerase (PARP) inhibitors (olaparib [8]), radioligand therapies (radium-223 [9] and lutetium-177-PSMA-617 [10]), and cancer vaccines (sipuleucel-T [11]), although they are not curative [3,12,13].

In recent years, the progressive evolution in the molecular characterization of cancer disease has led to the discovery of novel genomic, proteomic, and immunological biomarkers that transcend the traditional tumor classification approach based on histology [14]. Indeed, increased knowledge of multi-omics (genomics, transcriptomics, proteomics, and digital pathology) has guided the identification of several actionable driver mutations shared by different tumor histotypes [15]. Therefore, a new era in the development of anti-cancer agents has come of age, characterized by the pursuit of histology-agnostic, biomarker-driven therapies [16]. In parallel, a transformation in clinical trial design has occurred as a consequence of this changing perspective, with the introduction of basket and platform trials [17].

At present, the Food and Drug Administration (FDA) has approved six anti-cancer agents with histology-agnostic indications. In 2017, the immune checkpoint inhibitor (ICI) pembrolizumab was authorized for patients affected by metastatic solid tumors with mismatch-repair deficiency (dMMR) or high microsatellite instability (MSI-H) [18,19]. Subsequently, larotrectinib and entrectinib were approved to treat patients with advanced solid tumors harboring a neurotrophic tyrosine receptor kinase (NTRK) gene fusion; these approvals occurred in 2018 and 2019, respectively [20,21,22]. Three years after the first approval, the FDA expanded the tissue-agnostic indications for pembrolizumab to include patients with metastatic solid tumors with a high tumor mutational burden (TMB-H) [23,24]. Furthermore, dostarlimab, another ICI, was approved for patients with dMMR metastatic solid tumors in 2021 [25]. Finally, in 2022, the treatment combination of dabrafenib plus trametinib was granted accelerated approval for patients with advanced solid tumors harboring a BRAF V600E mutation [26].

### Objectives

The clinical trials that led to the approvals of the above-mentioned histology-agnostic drugs were conducted in biomarker-defined populations across several histological tumor types. However, only a small number of mCRPC patients were enrolled in those trials, providing few data on the activity of these drugs in this subset of patients [19,20,22,25]. This prompted us to critically review the scientific literature on the available evidence regarding the treatment efficacy of histology-agnostic drugs targeting dMMR/MSI-H, TMB-H, NTRK gene fusions, and the BRAF V600E mutation in mCRPC patients.

## 2. Methods

We searched MEDLINE for all relevant publications from its inception to 20 May 2022 without applied filters. The search strategy was established a priori by two authors (F.T. and G.M.I.) through discussion. Subsequently, two groups of authors conducted the literature search independently to evaluate the impact of the approved tissue-agnostic drugs on the treatment of mCRPC. Specifically, three authors (G.M.I., R.R., and M.C.) focused on both pembrolizumab for dMMR/MSI-H or TMB-H mCRPC and dostarlimab for dMMR mCRPC. In parallel, three additional authors evaluated the literature on both larotrectinib or entrectinib for NTRK-positive mCRPC and dabrafenib–trametinib in BRAF V600E-mutated mCRPC (S.R., S.G., and S.S.). A two-stage process of study selection was used in the literature search. First, all titles and abstracts were screened for potential relevance. Furthermore, the full texts of appropriate publications were retrieved and further assessed for eligibility. The agreement of both authors responsible for selection was required for exclusion at both stages. A consultation with two additional authors (M.R. and F.T.) was carried out for disagreements on study selection by consensus. Included publications were approved by all authors and, subsequently, uploaded to reference management software for further analyses. Finally, the authors achieved complete consensus on reporting the literature search results according to a biomarker-driven strategy: firstly, dMMR/MSI-H, then TMB-H, followed by NTRK gene fusions and, lastly, the BRAF V600E mutation.

## 3. Results

### 3.1. Mismatch-Repair Deficiency and High Microsatellite Instability

**Figure 1:**
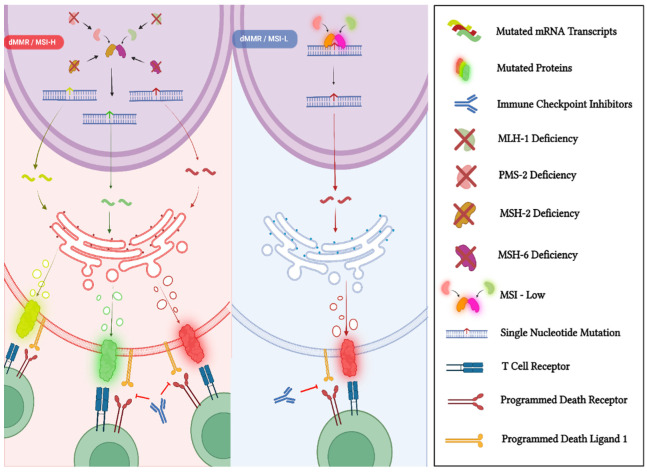
In normal cells, the MMR system guarantees DNA fidelity by detecting (MSH2/MSH6 complex) and repairing (MLH1/PMS2 complex) genetic mismatches that occurred during DNA replication. In contrast, dMMR/MSI-H tumor cells are not able to repair DNA mismatches in microsatellites and, consequently, are characterized by an accumulation of genomic alterations, which result in a higher quantity of neoantigens. This immunogenic phenotype is generated by a higher genomic mutational burden and provides dMMR/MSI-H tumors with an increased susceptibility to the reactivation of the anti-cancer response when treated with immune checkpoint inhibitors. “Created with BioRender.com”.

The DNA mismatch-repair system (MMR) represents a biological pathway that plays a crucial role in maintaining genomic stability [27]. It improves the fidelity of DNA replication by removing errors from newly synthesized DNA chains [28]. The principal genes involved in the MMR include MLH1, MSH2, MSH6, and PMS2, and the proteins of this complex system act as heterodimers (MSH2 binds to MSH6 and MLH1 binds to PMS2), correcting spontaneous base−base mispairs and small insertion−deletion loops that are mainly generated during DNA replication (Figure 1) [29]. However, when the MMR is deficient (also termed dMMR), it fails to recognize and repair these errors, resulting in an increased DNA mutational load [30]. The condition of dMMR is related to the loss of the expression of one of the MMR proteins; this may derive from inherited germline mutations in the coding genes, as in Lynch syndrome, or be caused by either the somatic mutation or the methylation of MMR genes [16]. The hallmark genomic feature associated with dMMR is microsatellite instability (MSI) [29]. Microsatellites consist of repetitive DNA sequences of between one and six nucleotides and are widely distributed throughout the whole genome and mostly located near the gene-coding regions [31]. They are particularly susceptible to mutations when the MMR is compromised, resulting in MSI status, which is characterized by a variation in the lengths of the microsatellite repeats [30]. According to the frequency of MSI, it is possible to distinguish between high MSI (MSI-H), low MSI (MSI-L), and microsatellite stability (MSS), although MSS-L and MSS tend to be classified as belonging to the same category [31].

A patient’s dMMR/MSI-H status can be determined through immunohistochemistry (IHC), polymerase chain reaction (PCR), and next-generation sequencing (NGS) techniques. The diagnosis of dMMR by IHC is established by the loss of expression of at least one MMR protein (MLH1, MSH2, MSH6, and PMS2) [31]. When the IHC results are indeterminate, further molecular analysis with PCR is recommended. In contrast, the evaluation of MSI-H status is generally performed with the PCR methodology by comparing tumor DNA samples with paired normal DNA [31]. At present, NGS panels represent alternative molecular tests to diagnose MSI-H status, with the advantage of evaluating both the TMB and the presence of additional actionable gene alterations concomitantly [32].

#### 3.1.1. dMMR/MSI-H Status in PCa

Several studies assessed the prevalence of dMMR/MSI-H in PCa patients and reported frequencies ranging between 3% and 5% (Table 1). The most commonly involved MMR genes were MSH2 and MSH6, mainly resulting from somatic mutational events [29]. At present, testing mCRPC patients for dMMR/MSI-H is highly recommended, and it should also be considered in the setting of both metastatic hormone-sensitive PCa and localized PCa [33,34]. Furthermore, molecular analyses should be performed on specimens obtained from a metastasis biopsy. Alternatively, if a biopsy is unsafe or unfeasible, dMMR/MSI-H status may be evaluated based on plasma-circulating tumor DNA (ctDNA), preferably collected at the time of biochemical or radiological progression [34]. When dMMR/MSI-H tumors are diagnosed, post-genetic counseling is also recommended to investigate a potential diagnosis of Lynch syndrome [34].

#### 3.1.2. Approval of Pembrolizumab and Dostarlimab for MSI-H/dMMR Solid Tumors

The genome of dMMR cancers is characterized by the presence of 10 to 100 times more mutations than mismatch repair–proficient (pMMR) tumors, regardless of the cell of origin [42]. This condition of “hypermutation” is associated with an increased expression of tumor neoantigens that facilitates immune recognition [43]. It has been hypothesized that this immunogenic phenotype, which is generated by a higher genomic mutational burden, provides these tumors with a higher susceptibility to the reactivation of the anti-cancer response when treated with immune checkpoint blockade [16]. In 2015, Le et al. reported the results of the KEYNOTE (KN)-016 trial, which aimed to evaluate the activity of pembrolizumab in a cohort of 41 patients with treatment-refractory metastatic carcinomas. This study showed an improvement in terms of the objective response rate (ORR) and median progression-free survival (mPFS) for dMMR colorectal cancer (CRC) and non-CRC patients compared to pMMR CRC patients [44]. In light of these results, the study was expanded to investigate the activity of pembrolizumab in 86 patients with twelve different metastatic dMMR tumor types. The results showed an ORR of 53% (95% Confidence Interval (CI), 42–64%), and complete responses (CRs) were achieved in 21% of patients [43]. Based on a combined analysis of five clinical trials (KN-164, KN-012, KN-028, KN-158, and KN-016), the FDA granted the approval of pembrolizumab as the first tissue-agnostic drug for solid tumors in 2017 [18]. In particular, the drug was approved with two indications: (i) the treatment of adult and pediatric patients affected by an unresectable or metastatic dMMR/MSI-H solid tumor that has progressed following prior treatments and where there are no satisfactory alternative treatment options; (ii) the treatment of unresectable or metastatic dMMR/MSI-H CRC patients that have progressed following treatment with fluoropyrimidine, oxaliplatin, and irinotecan [18]. The results from the dMMR cohort of the KN-158 trial were published in 2019. KN-158 was a non-randomized, phase 2 clinical trial aimed at evaluating the activity of pembrolizumab together with predictive biomarkers in patients with metastatic solid tumors. Among 233 enrolled patients affected by 27 tumor types and treated with pembrolizumab, the ORR was 34.3% (95% CI, 28.3–40.8%), and the mPFS was 4.1 months (95% CI, 2.4–4.9 months), after a median follow-up of 13.4 months [45].

In 2021, four years after pembrolizumab’s approval, the FDA approved dostarlimab, another programmed death protein-1 (PD-1) inhibitor, as a tissue-agnostic drug. In particular, dostarlimab was approved for adult patients with dMMR/MSI-H recurrent or advanced solid tumors that progressed on prior treatments and with no satisfactory alternative treatment options [25]. The authorization was granted based on the results of the non-randomized, multi-cohort GARNET trial in which dostarlimab was administered to 209 patients with dMMR recurrent or advanced solid tumors who had progressed following systemic therapy: the ORR was 41.6% (95% CI, 34.9–48.6%), with CR in 9.1% and partial response (PR) in 32.5% of patients [25].

#### 3.1.3. Pembrolizumab and Dostarlimab in MSI-H/dMMR mCRPC

Among the five studies in the above-mentioned combined analysis that led to the approval of pembrolizumab, only a few enrolled patients were affected by dMMR/MSI-H mCRPC. Notably, there were six patients (2.3% of the overall enrolled patients) in the KN-158 trial [45], and there was one patient (1.2%) in the KN-016 trial [43]. No mCRPC patients were enrolled in cohort F of the GARNET trial, which was reserved for non-endometrial metastatic MSI-H/dMMR solid tumors treated with dostarlimab [46].

In this context, while no publications are available for dostarlimab in dMMR/MSI-H mCRPC patients, several case reports (Table 2) and retrospective studies confirmed the efficacy of pembrolizumab in this specific subset of patients. In 2019, Antonarakis et al. published the first single-center retrospective study aiming to describe the clinical and histological features of mCRPC patients harboring deleterious MMR gene mutations, including the activity of standard therapies and PD-1 inhibitors [47]. Although thirteen patients with a deleterious MMR gene among MSH2, MSH6, MLH1, and PMS2 were identified, only four were treated with PD-1 blockade (two patients with pembrolizumab and two with nivolumab) as a fourth- to sixth-line treatment. Two patients achieved a PSA decline greater than 50% from baseline (PSA50), with a mPFS of 9 months (95% CI, 4–11 months). In addition, three patients achieved an objective soft-tissue response lasting for 3–9 months [47]. In the same year, Abida et al. published a single-center study to evaluate the prevalence of dMMR/MSI-H in a series of PCa patients who underwent a genomic profiling test based on NGS [38]. Among 1033 screened patients, 32 (3.1%) were diagnosed with dMMR/MSI-H PCa, and 11 (1.1%) mCRPC patients were treated with ICIs; in particular, treatment consisted of an anti-PD-1 agent in eight cases and an anti-programmed death ligand-1 (PD-L1) agent in three cases. Although the authors did not specify the ICI agent administered, six patients had a PSA50, with four achieving radiographic responses. At the time of publication, five of the six responders were still on therapy after as long as 89 weeks [38]. In 2020, Graham et al. published the results of a multicenter retrospective study describing the clinical and pathological characteristics of 27 dMMR/MSI-H mCRPC patients and their responses to PD-1 blockade. Data on PSA response were available for 15 out of the 17 patients who received pembrolizumab. Specifically, PSA50 occurred in eight (53%) patients, while the estimated PFS at six months was 64.1% (95% CI, 33.7–83.4%) [48]. Moreover, in the same year, Barata et al. published the first case series reporting the clinical activity of pembrolizumab for dMMR/MSI-H mCRPC assessed by a cfDNA assay. Among 14 mCRPC patients, 9 patients were treated with pembrolizumab after two lines of therapy. Four patients achieved PSA50 a median of 4 weeks after treatment initiation, including three patients with a PSA decline greater than 99%. The radiological response rate was 60%, with one CR and two PR among the five evaluable patients [49]. In 2021, Sena et al. published the results of a multicenter cohort of 65 dMMR mCRPC patients. Nineteen of those patients were treated with anti-PD-1 therapy: the PSA50 was 65%, and the mPFS was 24 weeks (95% CI, 16–54 weeks) [50].

### 3.2. Tumor Mutational Burden

**Figure 2:**
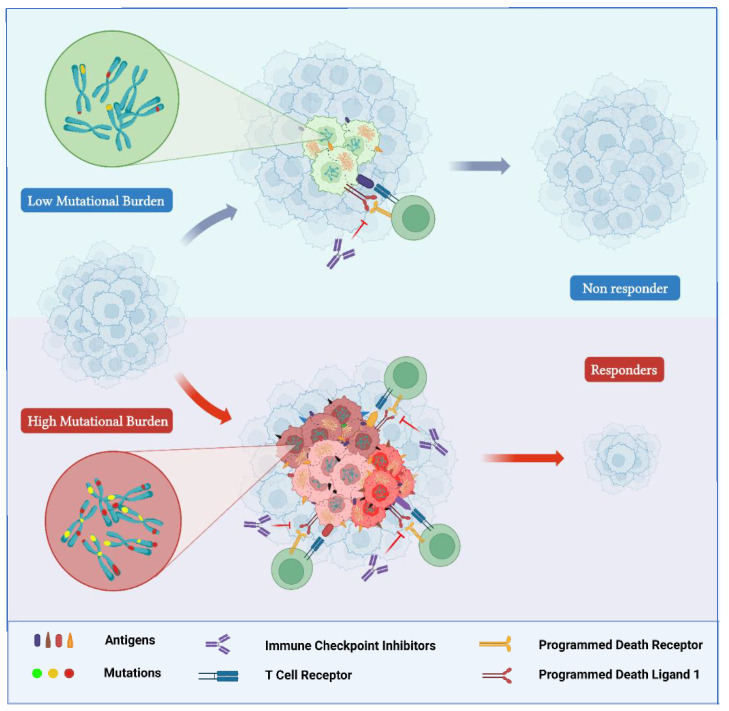
TMB-H tumors are characterized by higher levels of production of tumor-specific mutant epitopes that may function as neoantigens identified as “non-self” by the immune system. Hyper-mutated tumors (**bottom**) are more responsive than hypo-mutated tumors (**top**) to immune checkpoint inhibitors. “Created with BioRender.com”.

TMB quantifies the total number of somatic mutations per coding area of a tumor genome. It is generally determined as the number of mutations per megabase (mut/Mb) of the genome examined and is characterized by a highly variable pattern and distribution across different cancer types [58]. Indeed, an over 1000-fold difference in the total amount of somatic mutations has been detected between cancer types with the lowest TMB and those with the highest TMB, such as those associated with DNA environmental damage (exposure to tobacco smoke or ultraviolet rays) [59] or characterized by dMMR/MSI-H [60]. The presence of a high genomic burden of mutations is associated with a higher probability of producing tumor-specific mutant epitopes, which may potentially be recognized as “non-self” neoantigens by the immune system [59]. Actually, although not all mutations are responsible for generating tumor immunogenic peptides, it is acknowledged that their total number influences the overall amount of neoantigens potentially produced [59]. Therefore, it was hypothesized that cancers characterized by a high TMB would be more responsive to the increased activation of immune cells by treatment with an ICI (Figure 2) [61].

The initial studies aiming to establish a correlation between TMB and response to ICIs performed a TMB quantification based on whole-exome sequencing (WES) datasets [44]. Although WES permits a comprehensive measurement of TMB, it is an expensive and time-consuming technology to be used routinely in daily clinical practice. Therefore, multiple studies evaluated the opportunity to achieve equally accurate and clinically useful TMB estimates through NGS-based sequencing technologies [59]. At present, several NGS panels have been approved by the FDA to evaluate TMB-H status, and they can be distinguished in tissue- and liquid-biopsy-based tests. FoundationOne CDx (F1CDx) and MSK-IMPACT are two examples of tissue-based tests. The former identifies TMB by the number of base substitutions (including synonymous mutations) in the coding regions of targeted genes. In contrast, the latter tabulates non-synonymous mutations (NS) using tumor and germline DNA data. In addition, Guardant360 and FoundationOne Liquid CDx represent two liquid-biopsy tests that analyze cfDNA isolated from whole blood plasma specimens [62]. However, further studies are required to both standardize TMB assessment and determine the best cutoff for considering TMB as a predictive factor for response to ICIs [62].

#### 3.2.1. TMB-H Status in PCa

Several studies evaluated the TMB differently across multiple PCa cohorts, reporting TMB either as a load of NS mutations or as a load of any single-nucleotide variants. Furthermore, additional studies also described the rate of insertion–deletion mutations (indels) [63]. According to several retrospective studies, the TMB of locoregional PCa cohorts typically falls between 0.94 and 1.74 NS per megabase (NS/Mb), whereas it is typically higher in metastatic PCa cohorts, ranging from 2.08 to 4.1 NS/Mb [63]. Currently, TMB testing should be considered in patients affected by mCRPC [34].

#### 3.2.2. Approval of Pembrolizumab for TMB-H Solid Tumors

As mentioned above, multiple studies have shown a significant association between neoantigen production and immune-mediated clinical response [59]. This observation was confirmed by additional reports that documented a correlation between TMB-H (measured with different methods and cutpoints across studies) and the clinical benefit of ICIs [61]. Notably, the KN-158 trial investigated the activity of pembrolizumab in a prospectively planned retrospective analysis of patients with pre-treated unresectable or metastatic TMB-H solid tumors, defined as tissue TMB ≥ 10 mut/Mb with F1CDx [64]. Among 790 patients considered eligible for the analysis, only 102 were affected by TMB-H tumors. After a median follow-up of 37.1 months, the ORR was 29% (95% CI, 21%–39%) in the TMB-H group, with 4% CR and 25% PR. In contrast, the ORR in the non-TMB-H group was 6% (95% CI, 5–8%) [64]. In light of these findings, in 2020, the FDA approved pembrolizumab for the treatment of adult and pediatric patients with unresectable or metastatic TMB-H (≥10 mut/Mb) solid tumors (as determined by an FDA-approved test) that progressed on prior treatments and with no alternative therapeutic options [23].

#### 3.2.3. Pembrolizumab in TMB-H mCRPC

Although patients enrolled in the KN-158 trial were affected by a wide range of histological tumor types, no mCRPC patient was included in the prospectively planned retrospective analysis of metastatic TMB-H tumors [64]. As for dMMR/MSI-H, few publications evaluated the role of TMB in predicting ICI response in patients affected by mCRPC; mostly case reports (Table 2) and retrospective analyses are available.

In 2022, Graf et al. published a comparative effectiveness study to evaluate the treatment outcomes of mCRPC patients receiving ICIs compared to those receiving single-agent taxane chemotherapy; results were evaluated according to TMB status. A total of 741 patients received comprehensive genomic profiling, and 45 were treated with ICIs, while 696 were treated with single-agent taxane therapy. Among the patients treated with ICIs, 75.6% received pembrolizumab, 20% nivolumab, and 4.4% atezolizumab [65]. The results showed a worse median time to next therapy (TTNT) among patients with TMB < 10 mt/Mb receiving ICIs than for those receiving taxanes (2.4 vs. 4.1 months; hazard ratio (HR) 2.65; 95% CI, 1.78–3.95; *p* < 0.001). In contrast, for patients with TMB ≥ 10 mt/Mb, the administration of ICIs compared with taxanes was associated with more favorable outcomes, both in terms of median TTNT (8.0 vs. 2.4 months; HR 0.37; 95% CI, 0.15–0.87; *p* = 0.02) and overall survival (19.9 vs. 4.2 months; HR 0.23; 95% CI, 0.10–0.57; *p* = 0.001) [65]. Despite the study’s retrospective nature, it added validity to the TMB cutoff of 10 mt/Mb, suggesting that ICIs may be considered in TMB-H mCRPC patients for later lines of therapy. In this context, Sena et al. published, in 2021, a retrospective study on 65 dMMR mCRPC patients that aimed to evaluate the correlation of the number or proportion of tumor frameshift mutations with the response to anti-PD-1 therapy. As stated by the authors, considering the failure of TMB to predict responses to anti-PD-1 therapy in several types of tumors, including renal cell carcinoma and Hodgkin’s lymphoma, it was hypothesized that the T cell tolerance induced by the neoantigens caused by frameshift mutations would determine more robust antitumor activity [50]. TMB was calculated together with the frameshift mutation burden (FSB) and the frameshift mutation proportion (FSP) for the entire cohort of patients. The median TMB was 15 mut/Mb, the median FSB was 3.75 mut/Mb, and the median FSP was 0.17. The study results showed that FSP correlated more strongly than overall TMB with prolonged PFS and overall survival (OS) in terms of the anti-PD-1 treatment response [50].

### 3.3. Neurotrophic Tropomyosin Receptor Kinase Gene Fusions

**Figure 3:**
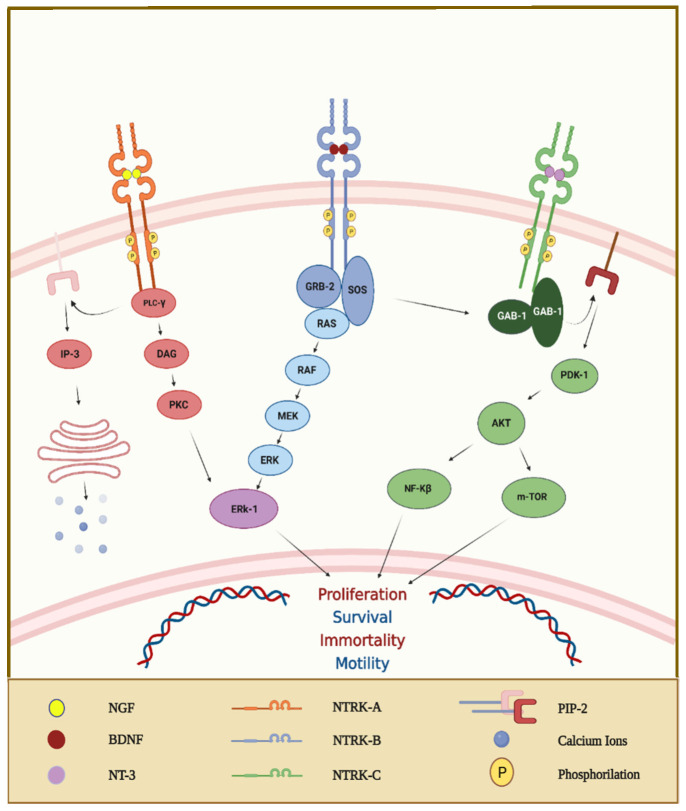
The interaction between ligands and TRK receptors generates TRK receptor dimerization, activating crosstalk between multiple intracellular signaling pathways involving PI3K and mitogen-activated protein kinase pathways. Abbreviations: protein kinase B (AKT); diacylglycerol (DAG); extracellular signal-regulated kinase (ERK-1); GRB2-associated binding protein-1 (GAB-1); growth factor receptor-bound protein-2 (GRB-2); inositol trisphosphate (IP-3); mitogen-activated protein kinase kinase (MEK); mammalian target of rapamycin (m-TOR); nuclear factor kinase-β (NF-Kβ); 3-phosphoinositide-dependent protein kinase-1 (PDK-1); protein kinase C (PKC); phospholipase C-γ (PLC-γ); rapidly accelerated fibrosarcoma (RAF); rat sarcoma virus (RAS); Son of sevenless (SOS). “Created with BioRender.com”.

The family of neurotrophin receptors is composed of three trans-membrane receptors (TRKA, TRKB, and TRKC) that are encoded by the NTRK1, NTRK2, and NTRK3 genes, respectively [66]. These receptors are composed of an intracellular domain, a transmembrane region, and an extracellular domain for ligand binding [67]. Specifically, TRKA is preferentially bound by the nerve growth factor (NGF), TRKB by the brain-derived neurotrophic factor (BDNF) and neurotrophin 4 (NT4), and finally, TRKC by neurotrophin 3 (NT3). The interaction between neurotrophins and their related receptors determines an intracellular cascade of signals leading to the activation of phosphatidylinositol 3-kinase (PI3K) and protein kinase B (AKT) or rat sarcoma virus (RAS) and extracellular signal-regulated kinase (ERK) (Figure 3) [68]. Initially, TRK receptors were identified in the central and peripheral nervous systems [69]. During embryogenesis, they regulate processes related to memory and the development of neuronal synapses, whereas they participate in both proprioception and a range of pain and appetite responses in adult life [68]. In recent years, multiple studies revealed that rearrangements of these genes, especially fusions, are detected in adult and pediatric tumors [69]. Indeed, NTRK gene fusions are frequent in some rare cancers, such as secretory breast carcinoma, congenital mesoblastic nephroma, and infantile fibrosarcoma; they also occur in other common tumors (CRC, melanoma, and non-small-cell lung cancer), although less frequently [69].

At present, several methods are available for identifying NTRK fusions: fluorescence in situ hybridization (FISH), reverse-transcriptase PCR (RT-PCR), and RNA- or DNA-based NGS. According to the recommendations published by the European Society for Medical Oncology (ESMO), two different methods should be used to investigate the presence of NTRK alterations depending on whether the tumor type is recognized as harboring highly recurrent NTRK gene fusions or not [70]. In the case of tumors with a high prevalence of NTRK alterations, FISH, RT-PCR, and targeted RNA NGS assays with specific probes can be used as confirmatory tests. Conversely, in the case of tumors with a low prevalence of NTRK fusions, the use of front-line NGS is recommended if a sequencing platform is available, in particular when testing RNA sequences. If the NGS test reveals NTRK gene fusions, an IHC test can be used to confirm the related protein expression. If a sequencing platform is not available, the use of IHC as a screening test is recommended, followed by NGS confirmation in case of a positive result [70].

#### 3.3.1. The NTRK Gene Fusions in PCa

In recent years, several studies aimed to describe the genomic landscape and prevalence of NTRK gene fusions in solid tumors, reporting frequencies of NTRK-positive PCa of less than 1%. In 2018, Ling et al. performed genomic profiling of over 3700 tumor samples from Chinese patients affected by solid tumors, identifying only one case of PCa harboring an IRF2BP2–NTRK1 translocation [71]. In 2019, Rosen et al. reported the analysis of genomic and clinical data concerning tumors harboring an NTRK fusion among more than 26.000 prospectively sequenced patients. Seventy-six cases (0.28%) with confirmed NTRK fusions were identified, mainly represented by salivary gland cancer, soft tissue sarcomas, and thyroid cancers. Although 1561 PCa patients were sequenced, none was identified as NTRK-fusion-positive [66]. In 2020, Forsythe et al. published a systematic review and meta-analysis analyzing NTRK gene fusion incidence among available studies published from 1987 to 2020. Rare tumors, such as secretory breast cancer, infantile fibrosarcoma, secretory salivary gland cancer, papillary thyroid carcinoma (pediatric), and congenital mesoblastic nephroma, were described as having the highest NTRK gene fusion frequencies (from 10 to 92.8%). On the contrary, the reported frequency for PCa was approximately 0% [72]. In 2021, Westphalen et al. published a retrospective study that evaluated the genomic landscape and prevalence of NTRK gene fusions in a large real-world database of comprehensive genomic profiling data (FoundationCORE). Among more than 295.000 cancer cases, salivary gland cancers (2.43%), soft tissue sarcomas (1.27%), and thyroid cancers (1.25%) were the most common tumors harboring NTRK gene fusions. In contrast, among the 9420 PCa cases analyzed, only 0.22% were reported as having an NTRK gene fusion [73]. Finally, Yeh et al. in 2019, reported on the case of a man affected by metastatic PCa at the diagnosis; PSA was 0.48 ng/mL, Gleason score was 9, and histology features included perineural and ganglional invasions. DNA and RNA–NGS showed PRPSAP1–NTRK3 fusion [74].

#### 3.3.2. Larotrectinib and Entrectinib in Tumors Harboring NTRK Fusion Genes

As mentioned above, several studies documented the presence of NTRK gene fusions in some tumors, and multiple drugs targeting the related proteins have been studied for the treatment of these malignancies. Among these compounds, two selective inhibitors of the TRK family of kinases are currently available: larotrectinib and entrectinib [75].

Larotrectinib is a highly selective tyrosine kinase inhibitor of all three TRK proteins, and its efficacy was first demonstrated in a combined analysis of three clinical trials published in 2018: a phase 1 trial (LOXO-TRK 14001), a phase 1–2 pediatric trial (SCOUT), and a phase 2 basket trial (NAVIGATE) [76]. Overall, fifty-five NTRK fusion-positive cancer patients aged between 4 months and 76 years were analyzed. Larotrectinib showed an overall response rate of 75% (95% CI, 61–85%), with 71% of the responses ongoing at one year of follow-up [76]. As a result, in November 2018, the FDA approved larotrectinib for the treatment of adult and pediatric patients who have solid tumors harboring an NTRK gene fusion, who are metastatic or ineligible for surgery, and who have progressed on prior treatments or have no satisfactory alternative therapy [20]. In 2020, Hong et al. published an expanded pooled efficacy analysis on 159 patients enrolled across the same three clinical trials. The results showed an ORR of 79% (95% CI, 72–85%) among the 153 patients evaluable for response, with 16% of patients achieving a CR, 63% a PR, 12% stable disease, and 6% progressive disease [77].

Entrectinib is a potent inhibitor of TRKA, TRKB, TRKC, ROS1, and anaplastic lymphoma kinase (ALK) and is also designed to cross the blood–brain barrier. Its efficacy was demonstrated in an integrated analysis of three phase 1–2 trials that included 54 adult patients with advanced or metastatic NTRK-fusion-positive solid tumors: 51 patients (94%) from STARTRK-2, 2 patients (4%) from STARTRK-1, and 1 patient (2%) from the ALKA-372–001 trial [78]. The analysis showed an objective response in 31 patients (57%; 95% CI, 43.2–70.8%): 4 (7%) patients had a CR and 27 (50%) a PR. Nine patients (17%) had stable disease as their best overall response to entrectinib. The response range was from 2.8 to 26 months, with 68% having a response duration greater than 6 months, after a median follow-up of 12.9 months [78]. In addition, an objective response of central nervous system metastases was observed in three of four patients with brain metastases, in the absence of brain radiotherapy [78]. As a result, in August 2019, the FDA approved entrectinib for the treatment of pediatric (older than 12 years) and adult patients affected by metastatic solid tumors harboring an NTRK gene fusion, who had progressed on prior treatments or have no satisfactory alternative therapy [21,22].

#### 3.3.3. Larotrectinib and Entrectinib in mCRPC

No mCRPC patients were enrolled in the studies that led to the approval of entrectinib [78], whereas only one patient was reported in the combined analysis by Hong et al. on larotrectinib efficacy [77]. Moreover, no further studies are yet available on patients affected by mCRPC treated with NTRK inhibitors.

### 3.4. BRAF V600E Mutation

**Figure 4:**
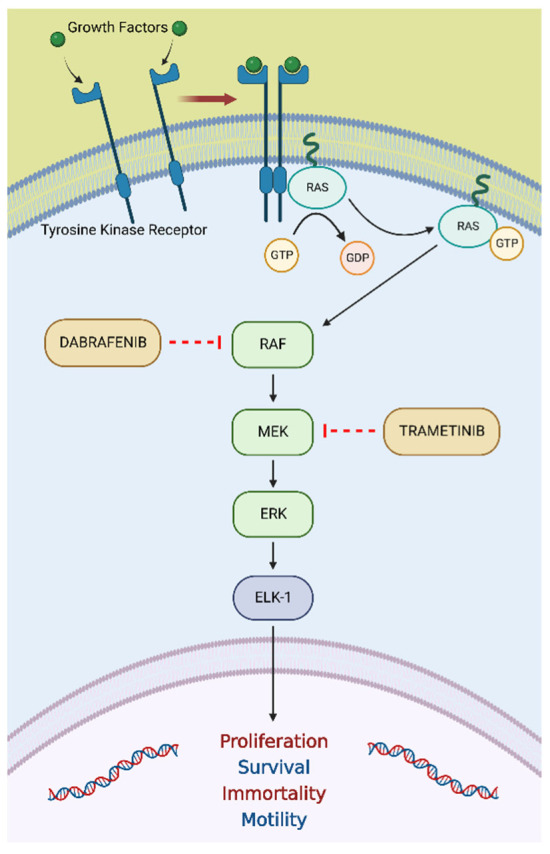
The RAS/RAF/MEK/ERK pathway is an essential molecular pathway among all MAPK signal transduction pathways and plays a crucial role in several cellular processes, including proliferation, differentiation, apoptosis, and stress responses. Abbreviations: ETS-like-1 (ELK-1); extracellular signal-regulated kinase (ERK); mitogen-activated protein kinase (MEK); rapidly accelerated fibrosarcoma (RAF); rat sarcoma virus (RAS). “Created with BioRender.com”.

BRAF (v-raf murine sarcoma viral oncogene homolog B1) is a protein kinase that belongs to the rapidly accelerated fibrosarcoma (RAF) family of serine/threonine kinases. It is involved in the mitogen-activated protein kinase (MAPK) cell signaling pathway, which transfers extracellular signals through the cells by a cascade of phosphorylation events (Figure 4) [79]. The activation of this molecular pathway results in a wide range of events related to cell growth, survival, and differentiation. Although three RAF kinases (ARAF, BRAF, and CRAF) play a physiological role in mammalian cells, BRAF is the most frequently altered kinase detected in a wide range of solid tumors and a subset of hematological malignancies [80]. Indeed, BRAF point mutations have been described in melanoma, non-Hodgkin’s lymphoma, CRC, papillary thyroid carcinoma, non-small-cell lung cancer, and gliomas [81]. Most of the BRAF mutations occur in the activation domain of the kinase, causing the permanent activation of BRAF and phosphorylation of MEK, independent of upstream activation by receptor tyrosine kinases or RAS. This results in the constitutive activation of ERK and, thus, in the promotion of cellular growth and evasion of apoptosis [82]. However, BRAF mutations can be distinguished into three different classes based on their effect on the activity of BRAF. Specifically, class 1 BRAF mutants are RAS-independent and act as an active monomer, whereas class 2 mutants function as an active dimer. These two classes of mutations determine the constitutive activation of BRAF independent of upstream growth stimuli. On the contrary, class 3 mutant BRAF proteins depend on RAS signaling for optimum activation [80]. Although almost 30 different BRAF mutations have been functionally characterized so far [80], the most common mutation is a class 1 missense mutation represented by the substitution of valine with glutamic acid at amino acid 600 (V600E). It has been detected in more than 90% of BRAF-mutated tumors [81].

At present, multiple methods have been developed to assess the presence of BRAF mutations, including IHC, PCR, RT-PCR, and NGS. However, in daily clinical practice, a sequential analysis of two methods is suggested, with the initial detection of BRAF mutations by IHC confirmed later by a molecular mutation testing technique [83].

#### 3.4.1. BRAF V600E Mutation in PCa

As mentioned above, the V600E is the most common activating mutation of BRAF, capable of generating uncontrolled cell growth and tumorigenesis by the hyper-activation of the MAPK pathway [84]. In recent years, a few retrospective studies evaluated the molecular status of BRAF in PCa patients. In 2009, Liu et al. published a study that aimed to evaluate the presence of BRAF mutations using DNA melting analysis with high-resolution technology on 93 PCa samples. No BRAF mutations were found in the examined cases [85]. The following year, Shen et al. assessed 121 PCa samples from a Chinese population for the presence of mutations, both at codons 12 or 13 of KRAS and at codon 600 of BRAF, using a mutant-enriched PCR-coupled sequencing method. While KRAS mutations were detected in 9.1% of patients, no BRAF mutations were detected in the examined cases [86]. In 2018, Jafarian et al. reported the results of a retrospective analysis of the BRAF molecular status of 100 samples of PCa. The BRAF V600E mutation was found in only four patients [87]. In addition to the previous data, several studies on PCa genomic profiling in the last decade reported further BRAF alterations, including mutations different from V600E, fusions, and amplification (Table 3).

#### 3.4.2. Dabrafenib plus Trametinib in Tumors Harboring a BRAF V600E Mutation

Increased knowledge of BRAF biology has led to the development of several targeted therapies able to inactivate its catalytic activity, including sorafenib, vemurafenib, and dabrafenib [80]. Beyond their clinical relevance, the development of these anti-cancer agents contributed to the further understanding of BRAF physiological regulation. Indeed, MEK inhibitors (including trametinib) started being investigated as an alternative to BRAF inhibitors, providing significant evidence that led to the strategy of RAF and MEK dual inhibition in BRAF V600E-mutated tumors [80].

In this context, two clinical trials were developed that aimed to investigate the combination treatment of dabrafenib and trametinib in adult cancer patients harboring a BRAF V600 mutation, regardless of tumor histology: the Rare Oncology Agnostic Research (ROAR) trial [95] and the National Cancer Institute’s Molecular Analysis for Therapy Choice (NCI-MATCH/EAY-131) trial [96]. The ROAR trial was a non-randomized, open-label, single-arm, phase 2 basket trial designed to evaluate the activity and safety of dabrafenib plus trametinib in patients with BRAF V600E-mutated rare cancers. In recent years, several publications showed the benefit of this combination in different subsets of patients enrolled in this study. Firstly, in 2018, Subbiah et al. published results regarding the activity of dabrafenib and trametinib in BRAF V600E-mutated anaplastic thyroid cancer (ATC) [97]. Sixteen ATC patients were enrolled, and an ORR of 69% (95% CI, 41–89%) was reported after a median follow-up of 47 weeks. An updated analysis on the same cohort of 36 ATC patients was published four years later, showing an ORR of 56% (95% CI, 38.1–72.1%) after a median follow-up of 11.1 months [98]. Secondly, results for patients with BRAF V600E-mutated biliary tract cancer were published in 2020. A total of 43 patients were enrolled, and an independent-reviewer-assessed overall response was achieved by 20 patients (47%; 95% CI, 31–62%) after a median follow-up of 10 months [99]. Furthermore, Wen et al. reported, in 2022, results on the use of dabrafenib plus trametinib in BRAF V600E-mutation-positive high-grade and low-grade glioma patients. In the high-grade glioma cohort, 45 patients (31 with glioblastoma) were enrolled, and an ORR of 33% (95% CI, 20–49%) was reported after a median follow-up was 12.7 months. In parallel, in the low-grade glioma cohort, 13 patients were enrolled, with an ORR of 69% (95% CI, 39–91%) after a median follow-up of 32.2 months [100]. The NCI-MATCH trial was a national master protocol trial launched in 2015 and designed to enroll cancer patients with actionable genomic alterations in 1 of more than 30 biomarker-selected treatment arms, accessing experimental therapies and drugs approved for other cancer histologies [101]. In 2020, Salama et al. published the results of the NCI-MATCH trial’s subprotocol H, which aimed to investigate the combination of dabrafenib and trametinib in patients with solid tumors, lymphomas, or multiple myeloma harboring a BRAF V600 mutation [102]. Among 35 patients enrolled in the study, 29 were included in the primary efficacy analysis. The ORR was 38% (90% CI, 22.9–54.9%), and the responses were observed in seven different tumor histologies. The above-mentioned data and the results provided by CTMT212X2101 (NCT02124772) trial [103] led to the approval of the first combination of drugs with an agnostic indication on 22 June 2022. Indeed, dabrafenib and trametinib were granted accelerated approval by the FDA for the treatment of adult and pediatric patients (older than six years of age) with unresectable or metastatic solid tumors harboring a BRAF V600E mutation, provided that the patient both progressed on prior treatment and had no satisfactory alternative therapeutic options; however, CRC patients were excluded [26]. This approval was also supported by the results of three other clinical trials that evaluated the efficacy of this combination in melanoma and non-small-cell lung cancer patients: COMBI-d [104], COMBI-v [105], and BRF113928 [106].

#### 3.4.3. Dabrafenib plus Trametinib in mCRPC Harboring a BRAF V600E Mutation

No mCRPC patients were enrolled in the above-mentioned clinical trials that led to the approval of dabrafenib and trametinib with an agnostic indication. In addition, no case reports or retrospective studies evaluated this combination in this subset of patients.

## 4. Discussion

Decades of cancer research have established that tumors originating from different tissues correspond to distinct clinical entities and may have different prognoses. Consequently, treatment algorithms for specific cancers have been traditionally derived from a histology-oriented approach [16]. However, precision medicine has recently gained an expanding, key role in the choice of cancer treatments [107]. Improved knowledge of the genomic alterations involved in oncogenesis and the advent of comprehensive genomic profiling technologies have paved the way for a new field of research focused on the development of histology-agnostic drugs [16]. Indeed, the identification of specific genomic events that are common to various malignancies and defined as “actionable” (that is, potentially responsive to targeted agents or immunotherapy) [108] led to the design of a new generation of “genome-based” cancer drugs that can be delivered regardless of tumor histology [107]. Since 2017, the FDA has approved six drugs with histology-agnostic indications (Table 4).

The advent of precision medicine has expanded the available therapeutic options in the management of genitourinary cancers [116,117]. Notably, the treatment paradigm of mCRPC has evolved rapidly in recent years, with the introduction of both new anti-cancer agents [4,5,6,7,8,9,10,11] and more sensitive imaging methods that led to the more accurate diagnosis and staging of this type of tumor [3]. However, mCRPC patients are still characterized by a poor prognosis, with an approximate median survival of only three years [3]. Further research is needed to develop new treatment strategies and investigate promising molecular pathways in this subset of patients while learning from the negative results of past clinical trials [118,119,120,121]. Therefore, we critically reviewed the scientific literature on the available evidence regarding the treatment efficacy of histology-agnostic drugs in this subset of patients.

PCa is a heterogeneous disease, with a complex interplay between inherent germline susceptibility, acquired somatic genomic alterations, and micro- and macroenvironmental factors being involved in its development [3]. At present, it is well recognized that the androgen receptor (AR) signaling pathway has a key role throughout the different stages of PCa. Indeed, AR pathways are altered in about 70% of mCRPC cases due to AR-dependent mechanisms [122], such as AR gene amplifications, mutations, splice variants, and AR overexpression [123]. However, other additional molecular pathways are commonly involved in mCRPC. Multiple studies showed the presence of germline mutations in DNA repair genes in about 12% of mCRPC patients, mainly affecting homologous recombination repair genes, including BRCA2, BRCA1, CHEK2, and ATM [122]. In addition, genomic alterations involving the PTEN–PI3K–AKT pathway, such as the loss of PTEN, an important cell cycle regulator associated with metastatic progression [122], are frequently observed in mCRPC [124].

Increasing knowledge of PCa molecular pathways has led to the development of several targeted therapies for mCRPC, such as ARSI, PARP-inhibitors, and AKT-inhibitors. In this context, our review highlighted the low frequency of both NTRK gene fusions and BRAF V600E mutations in mCRPC [66,72,73,85,86]. This may explain the cause of the absence of mCRPC patients from the clinical trials that led to the approval of entrectinib and dabrafenib–trametinib [26,77]. However, although these genomic alterations are rare in mCRPC, recent intriguing data emerged about their role in this subset of patients. Firstly, Bagherabadi et al., in 2022, showed that the downregulation of NTRK1 was associated with both a decrease in immune cell infiltration (such as T cell CD8+) and a poor prognosis in PCa patients, suggesting NTRK1 as a potential prognostic factor in this setting [125]. Indeed, the authors underlined how these results invite the further assessment of NTRK1 as a biomarker for PCa’s early diagnosis and prognosis, together with its role as a predictive factor for responses to NTRK inhibitors. Secondly, despite the rarity of BRAF V600E-mutated mCRPC cases, a few studies reported the presence of another BRAF mutation in mCRPC: BRAF K601E. Indeed, this mutation, though rare, appeared to be the most common BRAF-activating mutation in PCa [126]. Considering the initial evidence on the efficacy of dabrafenib plus trametinib in BRAF K601E-mutated melanoma and lung cancers [127,128], it would be interesting to evaluate this combination in mCRPC patients harboring this specific mutation in the future.

PCa is considered an immunologically “cold” tumor due to its immunosuppressive microenvironment with a low neoantigen load [129]. The PCa tumor immune microenvironment (TME) is characterized by the limited presence of tumor-infiltrating lymphocytes (TILs), represented mainly by CD4+ regulatory T cells with a restricted number of CD8+ cells. In addition, M2-polarized tumor-associated macrophages and myeloid-derived suppressor cells are also detectable in the PCa TME, with the latter producing IL-23, which has been shown to be involved in the regulation of castration resistance by sustaining AR signaling [130]. PCa cells are also characterized by a PTEN loss that interacts with the interferon-1 pathway, resulting in immunosuppression [130]. Although ICIs can elicit deep and durable responses in some patients affected by metastatic cancer, the ORR for ICI treatment obtained in mCRPC patients has been reported as 3% for patients with tumors without PD-L1 expression and 5% for those with PD-L1-expressing tumors [65]. However, the combination of chemotherapy with ICIs may represent an opportunity to improve anticancer immune responses by enhancing neoantigen presentation, stimulating proinflammatory cytokine secretion, and reducing suppressive immune cell populations. Two phase 2 trials investigating the combination of docetaxel plus nivolumab (CheckMate 9KD trial) [131] or plus pembrolizumab (KN-365, Cohort B) [132] in chemotherapy-naïve mCRPC patients showed promising clinical activity. In light of these results, two phase 3 trials are currently evaluating the efficacy of docetaxel combined with nivolumab [133] or pembrolizumab [134] in comparison with docetaxel plus placebo in men with mCRPC.

In this context, dMMR/MSI-H has proven to be an effective predictive factor of ICI response across multiple histological tumor types, including mCRPC. Our review reported the results of several retrospective studies and case reports that confirmed the efficacy of pembrolizumab in dMMR/MSI-H mCRPC patients. Nevertheless, open questions remain about the optimal sequence of treatments for this subset of patients and the identification of additional biomarkers predictive of pembrolizumab response. In this direction, our review confirmed the potential role of TMB as another useful biomarker for identifying mCRPC patients who could benefit from ICIs. In addition, although the optimal cutoff for TMB is a matter of debate, the recent study published by Graf et al. added validity to the TMB threshold of 10 mt/Mb identified by the FDA as the TMB lower limit for pembrolizumab administration [65].

Finally, although the advances in NGS genomic profiling technologies enabled us to enrich our knowledge of the genomic alterations involved in carcinogenesis, most of this information is of unknown clinical relevance [135]. In this direction, a working group of the ESMO developed the ESMO Scale for Clinical Actionability of Molecular Targets (ESCAT) [136], a comprehensive and reproducible score that aims to provide a systematic analysis of genomic alterations based on their level of scientific evidence and clinical relevance. Molecular targets are distinguished into different tiers (I–V and X), and assignment to a specific tier depends on the current level of evidence and, thus, can change over time based on newly available data [136]. However, despite the more frequent use of NGS panels to identify multiple actionable drivers simultaneously, their costs are still neither sustainable for many patients nor covered by some payers. In addition, medical oncologists should inform patients that the chance of finding an actionable target is low. In contrast, the approved tissue-agnostics are reserved for patients who progressed on prior lines of therapy and, thus, NGS panel represents a resource for those with no satisfactory alternative therapeutic options [62]. Finally, considering the previous pros and cons, it appears recommendable to routinely perform NGS-based genomic profiling tests to investigate both dMMR/MSI-H and TMB status, as well as the presence of NTRK fusion genes and the BRAF V600E mutation in mCRPC patients at the time of diagnosis.

### 4.1. Future Perspectives

In recent years, the advent of precision medicine rapidly reshaped the treatment of several solid tumors with the approval of multiple anticancer agents targeting genomic biomarkers shared by different tumor types [137]. In the near future, we believe that increased knowledge of immune profiling, transcriptomics, and proteomics will provide new means for the stratification of cancer patients and new biomarkers for treatment response. However, the availability of multiple approved treatments for relatively narrow populations of cancer patients is posing significant challenges for healthcare systems [138]. In this context, it is essential to implement the precision medicine paradigm in clinical practice by adapting the current infrastructures and reimbursement policies to enable access to these anticancer agents for patients [138].

### 4.2. Limitations

The present study has some limitations: (i) we did not perform a systematic revision of the literature, considering the narrative nature of this review; (ii) we discussed the search strategy and the two-stage study selection process before scrutinizing the available literature, but we did not design a written protocol as in systematic or scoping reviews; (iii) although we evaluated the included studies by examining both the manuscript and the supplementary materials, it was not possible to discriminate which anti-PD-1 agent was used in some publications. However, we decided to include them, considering the high probability that pembrolizumab was administered in this subset of patients compared with other ICIs.

## 5. Conclusions

The advent of tissue-agnostic therapies represents a milestone in the precision oncology era. At present, six different drugs have been approved by the FDA with tissue-agnostic indications: pembrolizumab (both for tumors with the dMMR/MSI-H phenotype and for tumors with the TMB-H phenotype), dostarlimab (for dMMR tumors), larotrectinib and entrectinib (for tumors harboring NTRK fusions) and the combination dabrafenib–trametinib (for BRAF V600E-mutated tumors). We critically reviewed the scientific literature regarding the treatment efficacy of the above-mentioned drugs in mCRPC patients. Although the available evidence is derived from retrospective studies and case reports, our review confirmed the efficacy of pembrolizumab in dMMR/MSI-H mCRPC. In contrast, few data are available for dostarlimab, larotrectinib, entrectinib, and dabrafenib–trametinib in this subset of patients. As a result, we believe that large, multi-institutional registries that collect real-world data on patients treated with the approved tissue-agnostic drugs will provide a better understanding of their therapeutic role in mCRPC patients.

## Figures and Tables

**Table 1:** List of studies that assessed the frequencies of somatic and germline mutations of MMR genes among PCa patients.

First Author	Year of Publication	Type of PCa	Number of Patients	Staging	Somatic dMMR		Germline dMMR	
					Frequency (n)	Gene(s) Involved	Frequency (n)	Gene(s) Involved
Robinson et al. [35]	2015	CRPC	150	Advanced	2.7% (3)	MLH1-MSH2	NA	NA
Pritchard et al. [36]	2016	NA	692	Advanced	NA	NA	0.6% (4)	MSH2-MSH6-PMS2
Guedes et al. [37]	2017	Mixed	1176	Localized/Advanced	1.2% (14)	MSH2-MSH6	0.3% (4)	MSH2
Abida et al. [38]	2018	CRPC	1033	Localized/Advanced	3.1% (32)	MLH1-MSH2-MSH6-PMS2	0.8% (8)	MSH2-MSH6-PMS2
Latham et al. [39]	2019	NA	1048	Localized/Advanced	5.6% (54)	NA	0.3% (3)	MSH2-PMS2
Nicolosi et al. [40]	2019	Mixed	3350	Localized/Advanced	1.7% (58)	MLH1-MSH2-MSH6-PMS2	NA	NA
Wu et al. [41]	2021	Mixed	246	Localized/Advanced	NA	NA	2.4% (6)	MSH2

Abbreviations: castration-resistant prostate cancer (CRPC); mismatch repair system deficiency (dMMR); not available (NA); number of patients (n); prostate cancer (PCa).

**Table 2:** List of case reports and case series on dMMR/MSH-H and TMB-H mCRPC patients treated with pembrolizumab.

Publication		Patient Characteristics					Treatments before Pembrolizumab		Treatment with Pembrolizumab				
First Author	Year	Age	Histotype of PCa (Gleason Score)	Staging at Diagnosis	MSI-H/dMMR	TMB	Treatments for Localized Disease *	Treatment for Metastatic Disease *	PSA before the First Cycle	Number of Cycles	Best PSA Response	Best Radiological Response	Outcome
Shimizu et al. [51]	2022	67	NA (5 + 4)	Localized	MSH2-MSH6 (IHC-Tissue)	61 mut/Mb (Tissue)	ADT	AA-RT-Doce-Caba	35.67 ng/mL	NA	Undetectable	PR	AwD
Ravindranathan et al. [52]	2021	51	ADC (5 + 5)	Advanced	MSI-H (NGS-ctDNA)	-	-	Doce-AA-Enza-Carbo/VP-16	39.9 ng/dL	12 **	Undetectable	CR	AwD
		81	ADC (4 + 4)	Advanced	MSI-H (NGS-ctDNA)	-	-	ADT + RT-Enza-AA-SipT-Doce-R/223	86.8 ng/mL	5 **	0.11 ng/mL	PR	AwD
Sena et al. [53]	2021	60	ADC (4 + 5)	Localized	MLH1 and PMS2 (IHC-Tissue)	25 mut/Mb (Tissue)	RT + ADT	SipT-AA-RT-Enza-Pembro-Doce	NA	NA	Reduction of 79% from baseline	PR	DoD
Fujiwara et al. [54]	2020	52	NA (4 + 4)	Localized	MSI-H (NA)	-	RPr-RT	AA-Enza-Doce-Caba	16.6 ng/mL	5 **	6.1 ng/mL	PR	AwD
Han et al. [55]	2019	75	ADC (5 + 5)	Advanced	MSI-H (NGS-ctDNA)	-	-	AA-Carbo/Doce-RT-Carbo/Caba	NA	6 **	Undetectable	PR	AwD
Manogue et al. [56]	2019	64	NA (4 + 5)	Advanced	MSH2 (PCR-Tissue)	40.9 mut/Mb (ctDNA)	-	ADT-AA-Doce	15 ng/dL	12	<0.01 ng/mL	CR	AwD
Costa et al. [57]	2019	85	NA (5 + 5)	Localized	MSI-H (NA -Tissue)	-	RT-RPr	ADT-AA	16 ng/mL	8	Undetectable	PR	NA

* Treatments were inserted in the table sequentially according to the therapeutic strategy adopted by the authors. ** Treatment was ongoing at the time of publication. Abbreviations: abiraterone acetate (AA); adenocarcinoma (ADC); alive with disease (Awd); androgen deprivation therapy (ADT); cabazitaxel (Caba); carboplatin (Carbo); complete response (CR); died of disease (DoD); docetaxel (Doce); enzalutamide (Enza); etoposide (VP-16); immunohistochemistry (IHC); mutations per megabase (mut/Mb); next-generation sequencing (NGS); not applicable (NA); partial response (PR); polymerase chain reaction (PCR); prostate cancer (PCa); prostate-specific antigen (PSA); radical prostatectomy (RPr); radiotherapy (RT); radium 223 (R/223); sipuleucel-T (SipT); tumor mutational burden (TMB).

**Table 3:** List of studies on PCa genomic profiling that reported molecular alterations in the BRAF gene.

First Author	Year of Publication	Type of PCa	Number of Patients Enrolled	Staging	BRAF Alteration		
					Mutation (n)	Fusion (n)	Amplification (n)
Cyrta et al. [88]	2022	CRPC	12	Advanced	K601E (1)	-	-
Alhamar et al. [89]	2020	Mixed	19	Mixed	-	FAM131A-BRAF (1)	-
					-	SND1-BRAF (1)	-
Kasajima et al. [90]	2020	HSPC	21	Localized	K601E (1)	-	-
Suh et al. [91]	2020	HSPC	20	Locally advanced	K601E (3)	-	-
Ikeda et al. [92]	2019	NA	67	NA	NA (1)	-	NA (2)
Barata et al. [93]	2018	Mixed	66	Advanced	-	-	NA (4)
Ateeq et al. [94]	2015	NA	121	NA	-	NA (1)	NA (2)

Abbreviations: castration-resistant prostate cancer (CRPC); family with sequence similarity 131 member A (FAM131A); hormone-sensitive prostate cancer (HSPC); not available (NA); number of patients (n); prostate cancer (PCa); staphylococcal nuclease and tudor domain containing 1 (SND1).

**Table 4:** List of FDA-approved drugs with histology-agnostic indications.

Anticancer Agent Class	Drug	Trade Name *(Pharma. Industry)*	Target	FDA Approval Date	Indication	Other Agencies’ Approvals (Date)	References
Immune Checkpoint Inhibitors *(Monoclonal Antibodies)*	Pembrolizumab	Keytruda *(Merck & Co.)*	PD-1	23 May 2017	Adult and pediatric patients affected by unresectable or metastatic MSI-H/dMMR solid tumors that progressed on prior treatments and have no satisfactory alternative treatment options or by MSI-H/dMMR CRC that progressed following therapy with fluoropyrimidine, oxaliplatin, and irinotecan.	PMDA (30 November 2018) EMA * (24 March 2022) **	[18,19,109,110,111]
				16 June 2020	Adult and pediatric patients affected by unresectable or metastatic TMB-H *** solid tumors, as determined by an FDA-approved test, that progressed on prior treatments and have no satisfactory alternative treatment options.	-	[23,24]
	Dostarlimab	Jemperli (*GlaxoSmithKline*)	PD-1	17 August 2021	Adult patients affected by dMMR recurrent or advanced solid tumors (as determined by an FDA-approved test) that have progressed on prior treatments and have no satisfactory alternative therapeutic options.	-	[25]
Targeted Therapies *(Small Molecules/Kinase Inhibitors)*	Larotrectinib	Vitrakvi *(Loxo Oncology Inc. and Bayer)*	NTRK	26 November 2018	Adult and pediatric patients with solid tumors harboring an NTRK gene fusion, without a known acquired resistance mutation, that are either metastatic or where surgical resection is likely to result in severe morbidity, and who have no satisfactory alternative treatments or whose cancer has progressed following treatment.	EMA (19 July 2019)	[20,21,22,23,24,25,26,27,28,29,30,31,32,33,34,35,36,37,38,39,40,41,42,43,44,45,46,47,48,49,50,51,52,53,54,55,56,57,58,59,60,61,62,63,64,65,66,67,68,69,70,71,72,73,74,75,76,77,78,79,80,81,82,83,84,85,86,87,88,89,90,91,92,93,94,95,96,97,98,99,100,101,102,103,104,105,106,107,108,109,110,111,112]
	Entrectinib	Rozlytrek *(Genentech Inc.)*	NTRK	15 August 2019	Adult and pediatric patients 12 years of age and older with solid tumors that have an NTRK gene fusion, without a known acquired resistance mutation, that are metastatic or where surgical resection is likely to result in severe morbidity, and who have progressed following treatment or have no satisfactory alternative therapy.	PMDA (3 June 2019) EMA (31 July 2020)	[21,22,113,114,115]
	Dabrafenib– Trametinib	Tafinlar–Mekinist (*Novartis*)	BRAF and MEK 1–2	22 June 2022	Adult and pediatric patients (older than six years of age) with unresectable or metastatic solid tumors harboring a BRAF V600E mutation who both progressed on prior treatment and have no satisfactory alternative therapeutic options, excluding CRC patients.	-	[26]

* The approval was related to one of the following dMMR/MSI-H tumors: unresectable or metastatic CRC; advanced or recurrent EC; unresectable or metastatic gastric, small intestine, or biliary cancers. ** Additional indications were approved on this date. *** Defined as ≥ 10 mutations per megabase. Abbreviations: colorectal cancer (CRC); endometrial carcinoma (EC); European Medicines Agency (EMA); U.S. Food and Drug Administration (FDA); high microsatellite instability/mismatch-repair deficiency (MSI-H/dMMR); mitogen-activated protein kinase kinase (MEK); mutations/megabase (mut/Mb); National Medical Products Administration (NMPA); neurotrophic tyrosine receptor kinase (NTRK); Pharmaceuticals and Medical Devices Agency (PMDA); programmed death protein-1 (PD-1); high tumor mutational burden (TMB-H).

## Data Availability

Not applicable.

## References

[1] Siegel R.L., Miller K.D., Fuchs H.E., Jemal A. (2022). Cancer Statistics, 2022. CA Cancer J. Clin..

[2] Cattrini C., España R., Mennitto A., Bersanelli M., Castro E., Olmos D., Lorente D., Gennari A. (2021). Optimal Sequencing and Predictive Biomarkers in Patients with Advanced Prostate Cancer. Cancers.

[3] Sandhu S., Moore C.M., Chiong E., Beltran H., Bristow R.G., Williams S.G. (2021). Prostate Cancer. Lancet.

[4] Tannock I.F., de Wit R., Berry W.R., Horti J., Pluzanska A., Chi K.N., Oudard S., Théodore C., James N.D., Turesson I. (2004). Docetaxel plus Prednisone or Mitoxantrone plus Prednisone for Advanced Prostate Cancer. N. Engl. J. Med..

[5] De Bono J.S., Oudard S., Ozguroglu M., Hansen S., Machiels J.-P., Kocak I., Gravis G., Bodrogi I., Mackenzie M.J., Shen L. (2010). Prednisone plus Cabazitaxel or Mitoxantrone for Metastatic Castration-Resistant Prostate Cancer Progressing after Docetaxel Treatment: A Randomised Open-Label Trial. Lancet.

[6] de Bono J.S., Logothetis C.J., Molina A., Fizazi K., North S., Chu L., Chi K.N., Jones R.J., Goodman O.B., Saad F. (2011). Abiraterone and Increased Survival in Metastatic Prostate Cancer. N. Engl. J. Med..

[7] Scher H.I., Fizazi K., Saad F., Taplin M.-E., Sternberg C.N., Miller K., de Wit R., Mulders P., Chi K.N., Shore N.D. (2012). Increased Survival with Enzalutamide in Prostate Cancer after Chemotherapy. N. Engl. J. Med..

[8] De Bono J., Mateo J., Fizazi K., Saad F., Shore N., Sandhu S., Chi K.N., Sartor O., Agarwal N., Olmos D. (2020). Olaparib for Metastatic Castration-Resistant Prostate Cancer. N. Engl. J. Med..

[9] Parker C., Nilsson S., Heinrich D., Helle S.I., O’Sullivan J.M., Fosså S.D., Chodacki A., Wiechno P., Logue J., Seke M. (2013). Alpha Emitter Radium-223 and Survival in Metastatic Prostate Cancer. N. Engl. J. Med..

[10] Sartor O., de Bono J., Chi K.N., Fizazi K., Herrmann K., Rahbar K., Tagawa S.T., Nordquist L.T., Vaishampayan N., El-Haddad G. (2021). Lutetium-177-PSMA-617 for Metastatic Castration-Resistant Prostate Cancer. N. Engl. J. Med..

[11] Kantoff P.W., Higano C.S., Shore N.D., Berger E.R., Small E.J., Penson D.F., Redfern C.H., Ferrari A.C., Dreicer R., Sims R.B. (2010). Sipuleucel-T Immunotherapy for Castration-Resistant Prostate Cancer. N. Engl. J. Med..

[12] Rizzo A., Mollica V., Cimadamore A., Santoni M., Scarpelli M., Giunchi F., Cheng L., Lopez-Beltran A., Fiorentino M., Montironi R. (2020). Is There a Role for Immunotherapy in Prostate Cancer?. Cells.

[13] Turco F., Tucci M., Angusti T., Parente A., Di Stefano R.F., Urban S., Pisano C., Samuelly A., Audisio A., Audisio M. (2022). Role of Radium-223 Discontinuation Due to Adverse Events in Castration-Resistant Prostate Cancer Patients. A Retrospective Monocentric Analysis. Tumori.

[14] Pestana R.C., Sen S., Hobbs B.P., Hong D.S. (2020). Histology-Agnostic Drug Development—Considering Issues beyond the Tissue. Nat. Rev. Clin. Oncol.

[15] Marshall J.L., Peshkin B.N., Yoshino T., Vowinckel J., Danielsen H.E., Melino G., Tsamardinos I., Haudenschild C., Kerr D.J., Sampaio C. (2022). The Essentials of Multiomics. Oncologist.

[16] Tarantino P., Mazzarella L., Marra A., Trapani D., Curigliano G. (2021). The Evolving Paradigm of Biomarker Actionability: Histology-Agnosticism as a Spectrum, Rather than a Binary Quality. Cancer Treat. Rev..

[17] Park J.J.H., Siden E., Zoratti M.J., Dron L., Harari O., Singer J., Lester R.T., Thorlund K., Mills E.J. (2019). Systematic Review of Basket Trials, Umbrella Trials, and Platform Trials: A Landscape Analysis of Master Protocols. Trials.

[18] FDA Grants Accelerated Approval to Pembrolizumab for First Tissue/Site Agnostic Indication. https://www.fda.gov/drugs/resources-information-approved-drugs/fda-grants-accelerated-approval-pembrolizumab-first-tissuesite-agnostic-indication.

[19] Marcus L., Lemery S.J., Keegan P., Pazdur R. (2019). FDA Approval Summary: Pembrolizumab for the Treatment of Microsatellite Instability-High Solid Tumors. Clin. Cancer Res..

[20] FDA Approves Larotrectinib for Solid Tumors with NTRK Gene Fusions. https://www.fda.gov/drugs/fda-approves-larotrectinib-solid-tumors-ntrk-gene-fusions.

[21] FDA Approves Entrectinib for NTRK Solid Tumors and ROS-1 NSCLC. https://www.fda.gov/drugs/resources-information-approved-drugs/fda-approves-entrectinib-ntrk-solid-tumors-and-ros-1-nsclc.

[22] Marcus L., Donoghue M., Aungst S., Myers C.E., Helms W.S., Shen G., Zhao H., Stephens O., Keegan P., Pazdur R. (2021). FDA Approval Summary: Entrectinib for the Treatment of NTRK Gene Fusion Solid Tumors. Clin. Cancer Res..

[23] FDA Approves Pembrolizumab for Adults and Children with TMB-H Solid Tumors. https://www.fda.gov/drugs/drug-approvals-and-databases/fda-approves-pembrolizumab-adults-and-children-tmb-h-solid-tumors.

[24] Marcus L., Fashoyin-Aje L.A., Donoghue M., Yuan M., Rodriguez L., Gallagher P.S., Philip R., Ghosh S., Theoret M.R., Beaver J.A. (2021). FDA Approval Summary: Pembrolizumab for the Treatment of Tumor Mutational Burden-High Solid Tumors. Clin. Cancer Res..

[25] FDA Grants Accelerated Approval to Dostarlimab-Gxly for dMMR Advanced Solid Tumors. https://www.fda.gov/drugs/resources-information-approved-drugs/fda-grants-accelerated-approval-dostarlimab-gxly-dmmr-advanced-solid-tumors.

[26] FDA Grants Accelerated Approval to Dabrafenib in Combination with Trametinib for Unresectable or Metastatic Solid Tumors with BRAF V600E Mutation. https://www.fda.gov/drugs/resources-information-approved-drugs/fda-grants-accelerated-approval-dabrafenib-combination-trametinib-unresectable-or-metastatic-solid.

[27] Li G.-M. (2008). Mechanisms and Functions of DNA Mismatch Repair. Cell Res..

[28] Jiricny J. (2006). The Multifaceted Mismatch-Repair System. Nat. Rev. Mol. Cell Biol..

[29] Graham L.S., Schweizer M.T. (2022). Mismatch Repair Deficiency and Clinical Implications in Prostate Cancer. Prostate.

[30] Pećina-Šlaus N., Kafka A., Salamon I., Bukovac A. (2020). Mismatch Repair Pathway, Genome Stability and Cancer. Front. Mol. Biosci..

[31] Li K., Luo H., Huang L., Luo H., Zhu X. (2020). Microsatellite Instability: A Review of What the Oncologist Should Know. Cancer Cell Int..

[32] Kok M., Chalabi M., Haanen J. (2019). How I Treat MSI Cancers with Advanced Disease. ESMO Open.

[33] EAU Prostate Cancer Guidelines. https://uroweb.org/guidelines/prostate-cancer.

[34] NCCN Prostate Cancer Guidelines Version 4.2022. https://www.nccn.org/guidelines/guidelines-detail?category=1&id=1459.

[35] Robinson D., Van Allen E.M., Wu Y.-M., Schultz N., Lonigro R.J., Mosquera J.-M., Montgomery B., Taplin M.-E., Pritchard C.C., Attard G. (2015). Integrative Clinical Genomics of Advanced Prostate Cancer. Cell.

[36] Pritchard C.C., Mateo J., Walsh M.F., De Sarkar N., Abida W., Beltran H., Garofalo A., Gulati R., Carreira S., Eeles R. (2016). Inherited DNA-Repair Gene Mutations in Men with Metastatic Prostate Cancer. N. Engl. J. Med..

[37] Guedes L.B., Antonarakis E.S., Schweizer M.T., Mirkheshti N., Almutairi F., Park J.C., Glavaris S., Hicks J., Eisenberger M.A., De Marzo A.M. (2017). MSH2 Loss in Primary Prostate Cancer. Clin. Cancer Res..

[38] Abida W., Cheng M.L., Armenia J., Middha S., Autio K.A., Vargas H.A., Rathkopf D., Morris M.J., Danila D.C., Slovin S.F. (2019). Analysis of the Prevalence of Microsatellite Instability in Prostate Cancer and Response to Immune Checkpoint Blockade. JAMA Oncol..

[39] Latham A., Srinivasan P., Kemel Y., Shia J., Bandlamudi C., Mandelker D., Middha S., Hechtman J., Zehir A., Dubard-Gault M. (2019). Microsatellite Instability Is Associated with the Presence of Lynch Syndrome Pan-Cancer. J. Clin. Oncol..

[40] Nicolosi P., Ledet E., Yang S., Michalski S., Freschi B., O’Leary E., Esplin E.D., Nussbaum R.L., Sartor O. (2019). Prevalence of Germline Variants in Prostate Cancer and Implications for Current Genetic Testing Guidelines. JAMA Oncol..

[41] Wu J., Wei Y., Pan J., Jin S., Gu W., Gan H., Zhu Y., Ye D.-W. (2021). Prevalence of Comprehensive DNA Damage Repair Gene Germline Mutations in Chinese Prostate Cancer Patients. Int. J. Cancer.

[42] Cancer Genome Atlas Network (2012). Comprehensive Molecular Characterization of Human Colon and Rectal Cancer. Nature.

[43] Le D.T., Durham J.N., Smith K.N., Wang H., Bartlett B.R., Aulakh L.K., Lu S., Kemberling H., Wilt C., Luber B.S. (2017). Mismatch Repair Deficiency Predicts Response of Solid Tumors to PD-1 Blockade. Science.

[44] Le D.T., Uram J.N., Wang H., Bartlett B.R., Kemberling H., Eyring A.D., Skora A.D., Luber B.S., Azad N.S., Laheru D. (2015). PD-1 Blockade in Tumors with Mismatch-Repair Deficiency. N. Engl. J. Med..

[45] Marabelle A., Le D.T., Ascierto P.A., Di Giacomo A.M., De Jesus-Acosta A., Delord J.-P., Geva R., Gottfried M., Penel N., Hansen A.R. (2020). Efficacy of Pembrolizumab in Patients with Noncolorectal High Microsatellite Instability/Mismatch Repair-Deficient Cancer: Results From the Phase II KEYNOTE-158 Study. J. Clin. Oncol..

[46] Safety and Efficacy of anti–PD-1 Antibody Dostarlimab in Patients (pts) with Mismatch Repair-Deficient (dMMR) Solid Cancers: Results from GARNET Study. https://ascopubs.org/doi/abs/10.1200/JCO.2021.39.3_suppl.9.

[47] Antonarakis E.S., Shaukat F., Isaacsson Velho P., Kaur H., Shenderov E., Pardoll D.M., Lotan T.L. (2019). Clinical Features and Therapeutic Outcomes in Men with Advanced Prostate Cancer and DNA Mismatch Repair Gene Mutations. Eur. Urol..

[48] Graham L.S., Montgomery B., Cheng H.H., Yu E.Y., Nelson P.S., Pritchard C., Erickson S., Alva A., Schweizer M.T. (2020). Mismatch Repair Deficiency in Metastatic Prostate Cancer: Response to PD-1 Blockade and Standard Therapies. PLoS ONE.

[49] Barata P., Agarwal N., Nussenzveig R., Gerendash B., Jaeger E., Hatton W., Ledet E., Lewis B., Layton J., Babiker H. (2020). Clinical Activity of Pembrolizumab in Metastatic Prostate Cancer with Microsatellite Instability High (MSI-H) Detected by Circulating Tumor DNA. J. Immunother. Cancer.

[50] Sena L.A., Fountain J., Isaacsson Velho P., Lim S.J., Wang H., Nizialek E., Rathi N., Nussenzveig R., Maughan B.L., Velez M.G. (2021). Tumor Frameshift Mutation Proportion Predicts Response to Immunotherapy in Mismatch Repair-Deficient Prostate Cancer. Oncologist.

[51] Shimizu K., Sano T., Mizuno K., Sunada T., Makita N., Hagimoto H., Goto T., Sawada A., Fujimoto M., Ichioka K. (2022). A Case of Microsatellite Instability-High Clinically Advanced Castration-Resistant Prostate Cancer Showing a Remarkable Response to Pembrolizumab Sustained over at Least 18 Months. Cold Spring Harb. Mol. Case Stud..

[52] Ravindranathan D., Russler G.A., Yantorni L., Drusbosky L.M., Bilen M.A. (2021). Detection of Microsatellite Instability via Circulating Tumor DNA and Response to Immunotherapy in Metastatic Castration-Resistant Prostate Cancer: A Case Series. Case Rep. Oncol..

[53] Sena L.A., Salles D.C., Engle E.L., Zhu Q., Tukachinsky H., Lotan T.L., Antonarakis E.S. (2021). Mismatch Repair-Deficient Prostate Cancer with Parenchymal Brain Metastases Treated with Immune Checkpoint Blockade. Cold Spring Harb. Mol. Case Stud..

[54] Fujiwara M., Komai Y., Yuasa T., Numao N., Yamamoto S., Fukui I., Yonese J. (2020). Pembrolizumab for a Patient with Metastatic Castration-Resistant Prostate Cancer with Microsatellite Instability-High. IJU Case Rep..

[55] Han H.J., Li Y.R., Roach M., Aggarwal R. (2020). Dramatic Response to Combination Pembrolizumab and Radiation in Metastatic Castration Resistant Prostate Cancer. Adv. Med. Oncol..

[56] Manogue C., Cotogno P., Ledet E., Lewis B., Wyatt A.W., Sartor O. (2019). Biomarkers for Programmed Death-1 Inhibition in Prostate Cancer. Oncologist.

[57] Costa L.B., Queiroz M.A., de Barbosa F.G., Nunes R.F., Marin J.F.G., Dzik C., Buchpiguel C.A. (2019). Pseudoprogression on PSMA PET Imaging of a MCRPC Patient under Anti-PD1 Treatment. Eur J. Nucl. Med. Mol. Imaging.

[58] Strickler J.H., Hanks B.A., Khasraw M. (2021). Tumor Mutational Burden as a Predictor of Immunotherapy Response: Is More Always Better?. Clin. Cancer Res..

[59] Fancello L., Gandini S., Pelicci P.G., Mazzarella L. (2019). Tumor Mutational Burden Quantification from Targeted Gene Panels: Major Advancements and Challenges. J. Immunother. Cancer.

[60] Chalmers Z.R., Connelly C.F., Fabrizio D., Gay L., Ali S.M., Ennis R., Schrock A., Campbell B., Shlien A., Chmielecki J. (2017). Analysis of 100,000 Human Cancer Genomes Reveals the Landscape of Tumor Mutational Burden. Genome Med..

[61] Huang T., Chen X., Zhang H., Liang Y., Li L., Wei H., Sun W., Wang Y. (2021). Prognostic Role of Tumor Mutational Burden in Cancer Patients Treated with Immune Checkpoint Inhibitors: A Systematic Review and Meta-Analysis. Front. Oncol..

[62] Weis L.N., Tolaney S.M., Barrios C.H., Barroso-Sousa R. (2021). Tissue-Agnostic Drug Approvals: How Does This Apply to Patients with Breast Cancer?. NPJ Breast Cancer.

[63] Ryan M.J., Bose R. (2019). Genomic Alteration Burden in Advanced Prostate Cancer and Therapeutic Implications. Front. Oncol..

[64] Marabelle A., Fakih M., Lopez J., Shah M., Shapira-Frommer R., Nakagawa K., Chung H.C., Kindler H.L., Lopez-Martin J.A., Miller W.H. (2020). Association of Tumour Mutational Burden with Outcomes in Patients with Advanced Solid Tumours Treated with Pembrolizumab: Prospective Biomarker Analysis of the Multicohort, Open-Label, Phase 2 KEYNOTE-158 Study. Lancet Oncol..

[65] Graf R.P., Fisher V., Weberpals J., Gjoerup O., Tierno M.B., Huang R.S.P., Sayegh N., Lin D.I., Raskina K., Schrock A.B. (2022). Comparative Effectiveness of Immune Checkpoint Inhibitors vs Chemotherapy by Tumor Mutational Burden in Metastatic Castration-Resistant Prostate Cancer. JAMA Netw. Open.

[66] Rosen E.Y., Goldman D.A., Hechtman J.F., Benayed R., Schram A.M., Cocco E., Shifman S., Gong Y., Kundra R., Solomon J.P. (2020). TRK Fusions Are Enriched in Cancers with Uncommon Histologies and the Absence of Canonical Driver Mutations. Clin. Cancer Res..

[67] Amatu A., Sartore-Bianchi A., Siena S. (2016). NTRK Gene Fusions as Novel Targets of Cancer Therapy across Multiple Tumour Types. ESMO Open.

[68] Chao M.V. (2003). Neurotrophins and Their Receptors: A Convergence Point for Many Signalling Pathways. Nat. Rev. Neurosci..

[69] Cocco E., Scaltriti M., Drilon A. (2018). NTRK Fusion-Positive Cancers and TRK Inhibitor Therapy. Nat. Rev. Clin. Oncol..

[70] Marchiò C., Scaltriti M., Ladanyi M., Iafrate A.J., Bibeau F., Dietel M., Hechtman J.F., Troiani T., López-Rios F., Douillard J.-Y. (2019). ESMO Recommendations on the Standard Methods to Detect NTRK Fusions in Daily Practice and Clinical Research. Ann. Oncol..

[71] Ling Q., Li B., Wu X., Wang H., Shen Y., Xiao M., Yang Z., Ma R., Chen D., Chen H. (2018). The Landscape of NTRK Fusions in Chinese Patients with Solid Tumor. Ann. Oncol..

[72] Forsythe A., Zhang W., Phillip Strauss U., Fellous M., Korei M., Keating K. (2020). A Systematic Review and Meta-Analysis of Neurotrophic Tyrosine Receptor Kinase Gene Fusion Frequencies in Solid Tumors. Adv. Med. Oncol..

[73] Westphalen C.B., Krebs M.G., Le Tourneau C., Sokol E.S., Maund S.L., Wilson T.R., Jin D.X., Newberg J.Y., Fabrizio D., Veronese L. (2021). Genomic Context of NTRK1/2/3 Fusion-Positive Tumours from a Large Real-World Population. NPJ Precis. Oncol..

[74] Yeh Y.A., Yang S., Constantinescu M., Chaudoir C., Tanner A., Henry M., Anderson S., Saldivar J.-S., Serkin F., Fazili T. (2019). Prostatic Adenocarcinoma with Novel NTRK3 Gene Fusion: A Case Report. Am. J. Clin. Exp. Urol..

[75] Lassen U. (2019). How I Treat NTRK Gene Fusion-Positive Cancers. ESMO Open.

[76] Drilon A., Laetsch T.W., Kummar S., DuBois S.G., Lassen U.N., Demetri G.D., Nathenson M., Doebele R.C., Farago A.F., Pappo A.S. (2018). Efficacy of Larotrectinib in TRK Fusion-Positive Cancers in Adults and Children. N. Engl. J. Med..

[77] Hong D.S., DuBois S.G., Kummar S., Farago A.F., Albert C.M., Rohrberg K.S., van Tilburg C.M., Nagasubramanian R., Berlin J.D., Federman N. (2020). Larotrectinib in Patients with TRK Fusion-Positive Solid Tumours: A Pooled Analysis of Three Phase 1/2 Clinical Trials. Lancet Oncol..

[78] Doebele R.C., Drilon A., Paz-Ares L., Siena S., Shaw A.T., Farago A.F., Blakely C.M., Seto T., Cho B.C., Tosi D. (2020). Entrectinib in Patients with Advanced or Metastatic NTRK Fusion-Positive Solid Tumours: Integrated Analysis of Three Phase 1–2 Trials. Lancet Oncol..

[79] Holderfield M., Deuker M.M., McCormick F., McMahon M. (2014). Targeting RAF Kinases for Cancer Therapy: BRAF-Mutated Melanoma and Beyond. Nat. Rev. Cancer.

[80] Zaman A., Wu W., Bivona T.G. (2019). Targeting Oncogenic BRAF: Past, Present, and Future. Cancers.

[81] Sholl L.M. (2020). A Narrative Review of BRAF Alterations in Human Tumors: Diagnostic and Predictive Implications. Precis. Cancer Med..

[82] Davies H., Bignell G.R., Cox C., Stephens P., Edkins S., Clegg S., Teague J., Woffendin H., Garnett M.J., Bottomley W. (2002). Mutations of the BRAF Gene in Human Cancer. Nature.

[83] Vanni I., Tanda E.T., Spagnolo F., Andreotti V., Bruno W., Ghiorzo P. (2020). The Current State of Molecular Testing in the BRAF-Mutated Melanoma Landscape. Front. Mol. Biosci..

[84] Sullivan R.J., Flaherty K.T. (2011). BRAF in Melanoma: Pathogenesis, Diagnosis, Inhibition, and Resistance. J. Skin Cancer.

[85] Liu T., Willmore-Payne C., Layfield L.J., Holden J.A. (2009). Lack of BRAF Activating Mutations in Prostate Adenocarcinoma: A Study of 93 Cases. Appl. Immunohistochem. Mol. Morphol..

[86] Shen Y., Lu Y., Yin X., Zhu G., Zhu J. (2010). KRAS and BRAF Mutations in Prostate Carcinomas of Chinese Patients. Cancer Genet. Cytogenet..

[87] Jafarian A.H., Mirshekar Nasirabadi K., Etemad S., Jafaripour M., Darijani M., Sheikhi M., Ayatollahi H., Shakeri S., Shams S.F., Davari S. (2018). Molecular Status of BRAF Mutation in Prostate Adenocarcinoma: The Analysis of 100 Cases in North-East of IRAN. Iran. J. Pathol..

[88] Cyrta J., Prandi D., Arora A., Hovelson D.H., Sboner A., Rodriguez A., Fedrizzi T., Beltran H., Robinson D.R., Gopalan A. (2022). Comparative Genomics of Primary Prostate Cancer and Paired Metastases: Insights from 12 Molecular Case Studies. J. Pathol..

[89] Alhamar M., Tudor Vladislav I., Smith S.C., Gao Y., Cheng L., Favazza L.A., Alani A.M., Ittmann M.M., Riddle N.D., Whiteley L.J. (2020). Gene Fusion Characterisation of Rare Aggressive Prostate Cancer Variants-Adenosquamous Carcinoma, Pleomorphic Giant-Cell Carcinoma, and Sarcomatoid Carcinoma: An Analysis of 19 Cases. Histopathology.

[90] Kasajima R., Yamaguchi R., Shimizu E., Tamada Y., Niida A., Tremmel G., Kishida T., Aoki I., Imoto S., Miyano S. (2020). Variant Analysis of Prostate Cancer in Japanese Patients and a New Attempt to Predict Related Biological Pathways. Oncol. Rep..

[91] Suh J., Jeong C.W., Choi S., Ku J.H., Kim H.H., Kim K.S., Kwak C. (2020). Targeted Next-Generation Sequencing for Locally Advanced Prostate Cancer in the Korean Population. Investig. Clin. Urol..

[92] Ikeda S., Elkin S.K., Tomson B.N., Carter J.L., Kurzrock R. (2019). Next-Generation Sequencing of Prostate Cancer: Genomic and Pathway Alterations, Potential Actionability Patterns, and Relative Rate of Use of Clinical-Grade Testing. Cancer Biol..

[93] Barata P.C., Mendiratta P., Heald B., Klek S., Grivas P., Sohal D.P.S., Garcia J.A. (2018). Targeted Next-Generation Sequencing in Men with Metastatic Prostate Cancer: A Pilot Study. Target. Oncol..

[94] Ateeq B., Kunju L.P., Carskadon S.L., Pandey S.K., Singh G., Pradeep I., Tandon V., Singhai A., Goel A., Amit S. (2015). Molecular Profiling of ETS and Non-ETS Aberrations in Prostate Cancer Patients from Northern India. Prostate.

[95] Efficacy and Safety of the Combination Therapy of Dabrafenib and Trametinib in Subjects with BRAF V600E-Mutated Rare Cancers. https://clinicaltrials.gov/ct2/show/NCT02034110.

[96] Targeted Therapy Directed by Genetic Testing in Treating Patients with Advanced Refractory Solid Tumors, Lymphomas, or Multiple Myeloma (The MATCH Screening Trial). https://clinicaltrials.gov/ct2/show/NCT02465060.

[97] Subbiah V., Kreitman R.J., Wainberg Z.A., Cho J.Y., Schellens J.H.M., Soria J.C., Wen P.Y., Zielinski C., Cabanillas M.E., Urbanowitz G. (2018). Dabrafenib and Trametinib Treatment in Patients with Locally Advanced or Metastatic BRAF V600-Mutant Anaplastic Thyroid Cancer. J. Clin. Oncol..

[98] Subbiah V., Kreitman R.J., Wainberg Z.A., Cho J.Y., Schellens J.H.M., Soria J.C., Wen P.Y., Zielinski C.C., Cabanillas M.E., Boran A. (2022). Dabrafenib plus Trametinib in Patients with BRAF V600E-Mutant Anaplastic Thyroid Cancer: Updated Analysis from the Phase II ROAR Basket Study. Ann. Oncol..

[99] Subbiah V., Lassen U., Élez E., Italiano A., Curigliano G., Javle M., de Braud F., Prager G.W., Greil R., Stein A. (2020). Dabrafenib plus Trametinib in Patients with BRAFV600E-Mutated Biliary Tract Cancer (ROAR): A Phase 2, Open-Label, Single-Arm, Multicentre Basket Trial. Lancet Oncol..

[100] Wen P.Y., Stein A., van den Bent M., De Greve J., Wick A., de Vos F.Y.F.L., von Bubnoff N., van Linde M.E., Lai A., Prager G.W. (2022). Dabrafenib plus Trametinib in Patients with BRAFV600E-Mutant Low-Grade and High-Grade Glioma (ROAR): A Multicentre, Open-Label, Single-Arm, Phase 2, Basket Trial. Lancet Oncol..

[101] Murciano-Goroff Y.R., Drilon A., Stadler Z.K. (2021). The NCI-MATCH: A National, Collaborative Precision Oncology Trial for Diverse Tumor Histologies. Cancer Cell.

[102] Salama A.K.S., Li S., Macrae E.R., Park J.-I., Mitchell E.P., Zwiebel J.A., Chen H.X., Gray R.J., McShane L.M., Rubinstein L.V. (2020). Dabrafenib and Trametinib in Patients with Tumors with BRAFV600E Mutations: Results of the NCI-MATCH Trial Subprotocol H. J. Clin. Oncol..

[103] Study to Investigate Safety, Pharmacokinetic (PK), Pharmacodynamic (PD) and Clinical Activity of Trametinib in Subjects with Cancer or Plexiform Neurofibromas and Trametinib in Combination with Dabrafenib in Subjects with Cancers Harboring V600 Mutations. https://clinicaltrials.gov/ct2/show/NCT02124772.

[104] Robert C., Karaszewska B., Schachter J., Rutkowski P., Mackiewicz A., Stroiakovski D., Lichinitser M., Dummer R., Grange F., Mortier L. (2015). Improved Overall Survival in Melanoma with Combined Dabrafenib and Trametinib. N. Engl. J. Med..

[105] Robert C., Grob J.J., Stroyakovskiy D., Karaszewska B., Hauschild A., Levchenko E., Chiarion Sileni V., Schachter J., Garbe C., Bondarenko I. (2019). Five-Year Outcomes with Dabrafenib plus Trametinib in Metastatic Melanoma. N. Engl. J. Med..

[106] Planchard D., Smit E.F., Groen H.J.M., Mazieres J., Besse B., Helland Å., Giannone V., D’Amelio A.M., Zhang P., Mookerjee B. (2017). Dabrafenib plus Trametinib in Patients with Previously Untreated BRAFV600E-Mutant Metastatic Non-Small-Cell Lung Cancer: An Open-Label, Phase 2 Trial. Lancet Oncol..

[107] Deininger M., Buchdunger E., Druker B.J. (2005). The Development of Imatinib as a Therapeutic Agent for Chronic Myeloid Leukemia. Blood.

[108] Carr T.H., McEwen R., Dougherty B., Johnson J.H., Dry J.R., Lai Z., Ghazoui Z., Laing N.M., Hodgson D.R., Cruzalegui F. (2016). Defining Actionable Mutations for Oncology Therapeutic Development. Nat. Rev. Cancer.

[109] EMA Recommends Extension of Indications for Pembrolizumab to MSI-H or dMMR Cancers and to Metastatic Cervical Cancer with PD-L1 CPS ≥1. https://www.esmo.org/oncology-news/ema-recommends-extension-of-indications-for-pembrolizumab-to-msi-h-or-dmmr-cancers-and-to-metastatic-cervical-cancer-with-pd-l1-cps-1.

[110] Trullas A., Delgado J., Genazzani A., Mueller-Berghaus J., Migali C., Müller-Egert S., Zander H., Enzmann H., Pignatti F. (2021). The EMA Assessment of Pembrolizumab as Monotherapy for the First-Line Treatment of Adult Patients with Metastatic Microsatellite Instability-High or Mismatch Repair Deficient Colorectal Cancer. ESMO Open.

[111] Pharmaceutical Evaluation Division, Pharmaceutical Safety and Environmental Health Bureau Ministry of Health, Labour and Welfare Pembrolizumab. https://www.pmda.go.jp/files/000231921.pdf.

[112] Vitrakvi (Larotrectinib). https://www.ema.europa.eu/en/medicines/human/EPAR/vitrakvi.

[113] Rozlytrek (Entrectinib). https://www.ema.europa.eu/en/medicines/human/EPAR/rozlytrek.

[114] Ardini E., Siena S. (2020). Entrectinib Approval by EMA Reinforces Options for ROS1 and Tumour Agnostic NTRK Targeted Cancer Therapies. ESMO Open.

[115] Pharmaceutical Evaluation Division, Pharmaceutical Safety and Environmental Health Bureau Ministry of Health, Labour and Welfare Entrectinib. https://www.pmda.go.jp/files/000232794.pdf.

[116] Pederzoli F., Bandini M., Marandino L., Ali S.M., Madison R., Chung J., Ross J.S., Necchi A. (2020). Targetable Gene Fusions and Aberrations in Genitourinary Oncology. Nat. Rev. Urol..

[117] Iannantuono G.M., Riondino S., Sganga S., Roselli M., Torino F. (2022). Activity of ALK Inhibitors in Renal Cancer with ALK Alterations: A Systematic Review. Int. J. Mol. Sci..

[118] Sayegh N., Swami U., Agarwal N. (2022). Recent Advances in the Management of Metastatic Prostate Cancer. JCO Oncol. Pract..

[119] Cereda V., Formica V., Massimiani G., Tosetto L., Roselli M. (2014). Targeting Metastatic Castration-Resistant Prostate Cancer: Mechanisms of Progression and Novel Early Therapeutic Approaches. Expert Opin. Investig. Drugs.

[120] Ioannidou E., Moschetta M., Shah S., Parker J.S., Ozturk M.A., Pappas-Gogos G., Sheriff M., Rassy E., Boussios S. (2021). Angiogenesis and Anti-Angiogenic Treatment in Prostate Cancer: Mechanisms of Action and Molecular Targets. Int. J. Mol. Sci..

[121] Cereda V., Formica V., Roselli M. (2018). Issues and Promises of Bevacizumab in Prostate Cancer Treatment. Expert Opin. Biol..

[122] Devlies W., Eckstein M., Cimadamore A., Devos G., Moris L., Van den Broeck T., Montironi R., Joniau S., Claessens F., Gevaert T. (2020). Clinical Actionability of the Genomic Landscape of Metastatic Castration Resistant Prostate Cancer. Cells.

[123] Ku S.-Y., Gleave M.E., Beltran H. (2019). Towards Precision Oncology in Advanced Prostate Cancer. Nat. Rev. Urol..

[124] Abida W., Cyrta J., Heller G., Prandi D., Armenia J., Coleman I., Cieslik M., Benelli M., Robinson D., Van Allen E.M. (2019). Genomic Correlates of Clinical Outcome in Advanced Prostate Cancer. Proc. Natl. Acad. Sci. USA.

[125] Bagherabadi A., Hooshmand A., Shekari N., Singh P., Zolghadri S., Stanek A., Dohare R. (2022). Correlation of NTRK1 Downregulation with Low Levels of Tumor-Infiltrating Immune Cells and Poor Prognosis of Prostate Cancer Revealed by Gene Network Analysis. Genes.

[126] Steinwald P., Ledet E., Sartor O. (2020). Eradication of BRAF K601E Mutation in Metastatic Castrate-Resistant Prostate Cancer Treated with Cabazitaxel and Carboplatin: A Case Report. Clin. Genitourin. Cancer.

[127] Su P.-L., Lin C.-Y., Chen Y.-L., Chen W.-L., Lin C.-C., Su W.-C. (2021). Durable Response to Combined Dabrafenib and Trametinib in a Patient with BRAF K601E Mutation-Positive Lung Adenocarcinoma: A Case Report. JTO Clin. Res. Rep..

[128] Rogiers A., Thomas D., Vander Borght S., van den Oord J.J., Bechter O., Dewaele M., Rambow F., Marine J.-C., Wolter P. (2019). Dabrafenib plus Trametinib in BRAF K601E-Mutant Melanoma. Br. J. Derm..

[129] Venkatachalam S., McFarland T.R., Agarwal N., Swami U. (2021). Immune Checkpoint Inhibitors in Prostate Cancer. Cancers.

[130] Rebuzzi S.E., Rescigno P., Catalano F., Mollica V., Vogl U.M., Marandino L., Massari F., Pereira Mestre R., Zanardi E., Signori A. (2022). Immune Checkpoint Inhibitors in Advanced Prostate Cancer: Current Data and Future Perspectives. Cancers.

[131] Fizazi K., González Mella P., Castellano D., Minatta J.N., Rezazadeh Kalebasty A., Shaffer D., Vázquez Limón J.C., Sánchez López H.M., Armstrong A.J., Horvath L. (2022). Nivolumab plus Docetaxel in Patients with Chemotherapy-Naïve Metastatic Castration-Resistant Prostate Cancer: Results from the Phase II CheckMate 9KD Trial. Eur. J. Cancer.

[132] Yu E.Y., Kolinsky M.P., Berry W.R., Retz M., Mourey L., Piulats J.M., Appleman L.J., Romano E., Gravis G., Gurney H. (2022). Pembrolizumab Plus Docetaxel and Prednisone in Patients with Metastatic Castration-Resistant Prostate Cancer: Long-Term Results from the Phase 1b/2 KEYNOTE-365 Cohort B Study. Eur. Urol..

[133] Drake C.G., Saad F., Clark W.R., Ciprotti M., Sharkey B., Subudhi S.K., Fizazi K. (2020). 690TiP A Phase III, Randomized, Double-Blind Trial of Nivolumab or Placebo Combined with Docetaxel for Metastatic Castration-Resistant Prostate Cancer (MCRPC; CheckMate 7DX). Ann. Oncol..

[134] Petrylak D.P., Ratta R., Gafanov R., Facchini G., Piulats J.M., Kramer G., Flaig T.W., Chandana S.R., Li B., Burgents J. (2021). KEYNOTE-921: Phase III Study of Pembrolizumab plus Docetaxel for Metastatic Castration-Resistant Prostate Cancer. Future Oncol..

[135] Wolff L., Kiesewetter B. (2022). Applicability of ESMO-MCBS and ESCAT for Molecular Tumor Boards. memo Mag. Eur. Med. Oncol..

[136] Mateo J., Chakravarty D., Dienstmann R., Jezdic S., Gonzalez-Perez A., Lopez-Bigas N., Ng C.K.Y., Bedard P.L., Tortora G., Douillard J.-Y. (2018). A Framework to Rank Genomic Alterations as Targets for Cancer Precision Medicine: The ESMO Scale for Clinical Actionability of Molecular Targets (ESCAT). Ann. Oncol..

[137] Adashek J.J., Subbiah V., Kurzrock R. (2021). From Tissue-Agnostic to N-of-One Therapies: (R)Evolution of the Precision Paradigm. Trends Cancer.

[138] Mateo J., Steuten L., Aftimos P., André F., Davies M., Garralda E., Geissler J., Husereau D., Martinez-Lopez I., Normanno N. (2022). Delivering Precision Oncology to Patients with Cancer. Nat. Med..

